# First Report of Fatal Infection Caused by Community-acquired Methicillin-resistant *Staphylococcus aureus* USA300 Clone in a Collegiate Athlete

**DOI:** 10.31662/jmaj.2019-0054

**Published:** 2020-01-09

**Authors:** Ryohei Yokomori, Junya Tsurukiri, Mariko Moriya, Hiroshi Yamanaka, Takehito Kobayashi, Hidemasa Nakaminami, Shunsuke Takadama, Norihisa Noguchi, Tetsuya Matsumoto, Takao Arai

**Affiliations:** 1 Emergency and Critical Care Medicine, Tokyo Medical University Hachioji Medical Center, Tokyo, Japan; 2 Department of Infection Prevention and Control, Tokyo Medical University Hachioji Medical Center, Tokyo, Japan; 3 Department of Microbiology, Tokyo Medical University, Tokyo, Japan; 4 Department of Microbiology, School of Pharmacy, Tokyo University of Pharmacy and Life Sciences, Tokyo, Japan; 5 Department of Medicine, International University of Health and Welfare, Narita, Japan

**Keywords:** community-acquired methicillin-resistant *Staphylococcus aureus*, USA300 clone, sepsis, bacteremia, acute respiratory distress syndrome

## Abstract

Community-acquired methicillin-resistant *Staphylococcus aureus* (CA-MRSA) is prevalent around the world and is a causative agent of skin and soft tissue infections in healthy individuals. Particularly, Panton–Valentine leukocidin (PVL)-positive CA-MRSA strains occasionally cause life-threatening infections, such as septic pulmonary emboli (SPE) and infectious endocarditis. However, severe infections caused by PVL-positive CA-MRSA strains have rarely been reported in Japan. For the first time, this study reports the case of a 20-year-old Japanese college athlete with life-threatening PVL-positive CA-MRSA USA300 clone infection, including sepsis, SPE, and skin and soft tissue infections with iliofemoral deep venous thrombosis.

## Introduction

USA300 clone is one of the highest pathogenic and global epidemic community-acquired methicillin-resistant *Staphylococcus aureus* (CA-MRSA) clones and carries the Panton–Valentine leukocidin (PVL) genes and arginine catabolic mobile element. It is a sequence-type (ST) 8-staphylococcal cassette chromosome (SCC) *mec* type IV (ST8-IV) ^(1)^. PVL targets polymorphonuclear leukocytes and macrophages and induces cell death via necrosis or apoptosis ^(2)^. Therefore, PVL-positive CA-MRSA causes severe diseases, such as skin and soft tissue infections (SSTIs), necrotizing pneumonia, and bacteremia ^(3)^. Recently in Japan, USA300 clone infection has been increasing in not only hospitals but also in community settings ^(4)^. Here we report the case of a Japanese college athlete with septic pulmonary emboli (SPE) secondary to infectious iliofemoral deep venous thrombosis (DVT) and abscesses caused by the USA300 clone.

## Case Report

**Figure 1.**
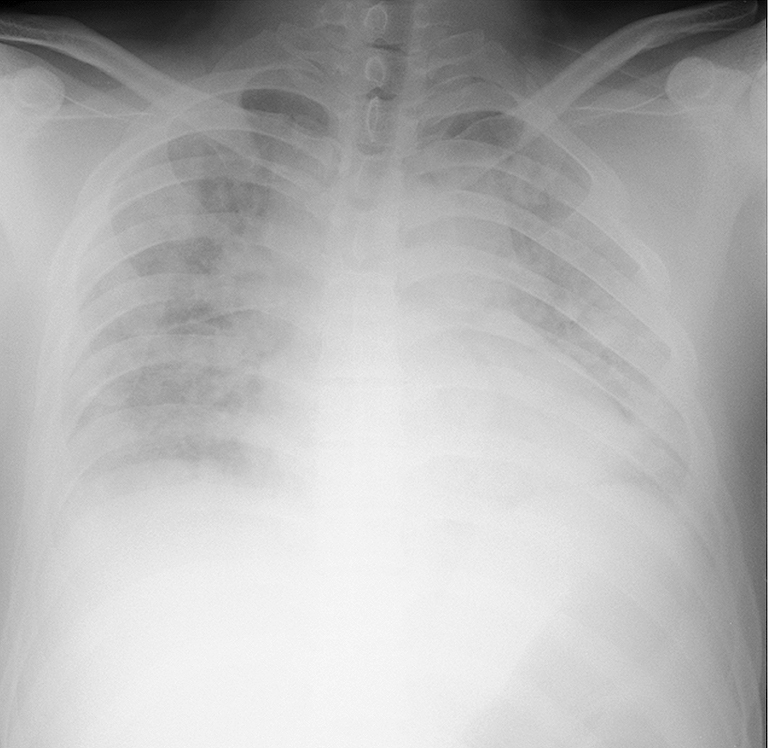
Chest radiography revealed bilateral alveolar shadows.

**Figure 2.**
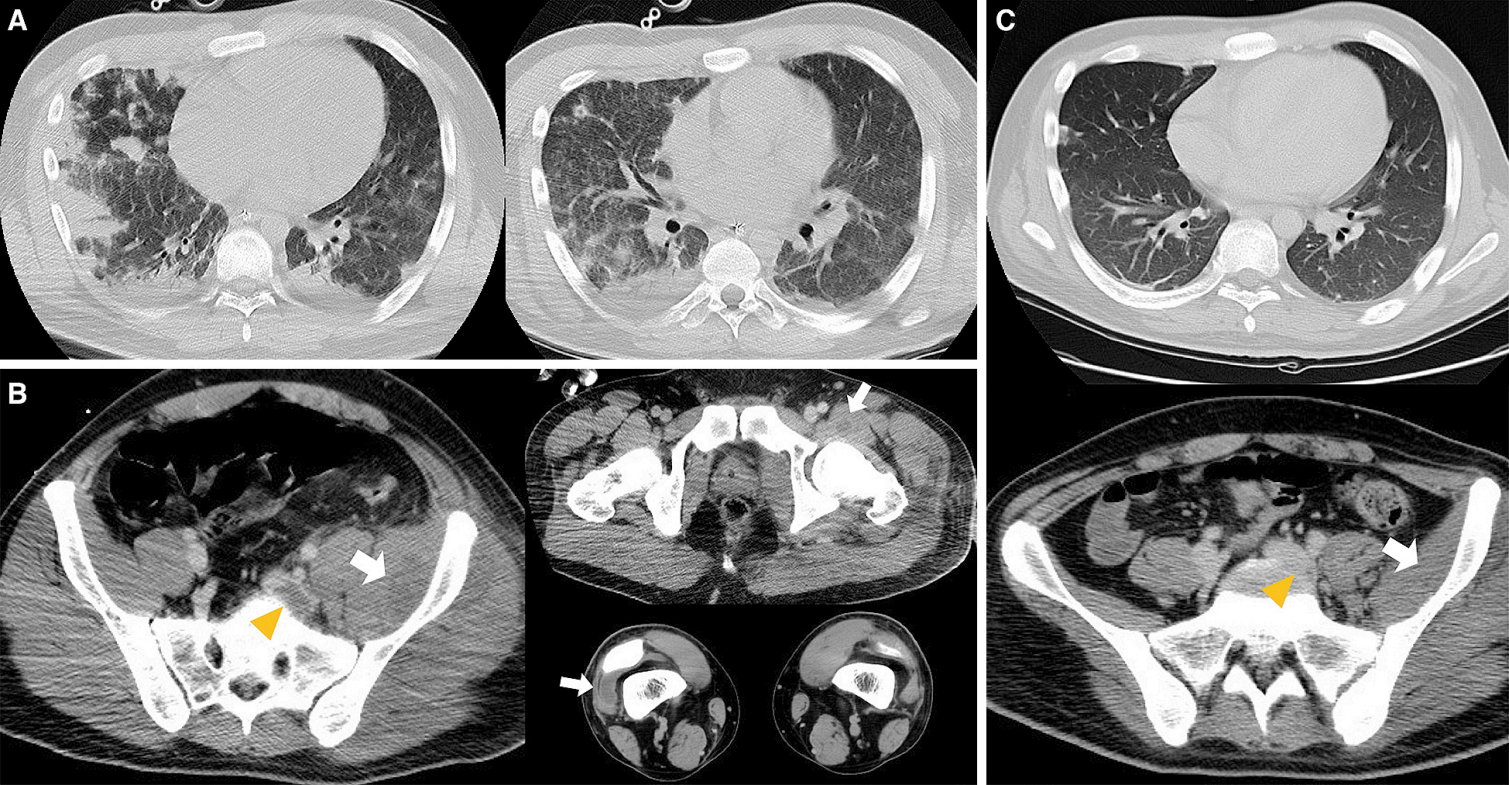
A) Contrast-enhanced computed tomography of the chest revealed septic pulmonary emboli. B) Contrast-enhanced computed tomography revealed left iliofemoral thrombosis (arrow head) and skin and soft tissue infection of the hip and left thigh with abscesses (arrow). C) CT after 1 month revealed the resolution of the iliofemoral deep venous thrombosis (arrow head).

**Figure 3.**
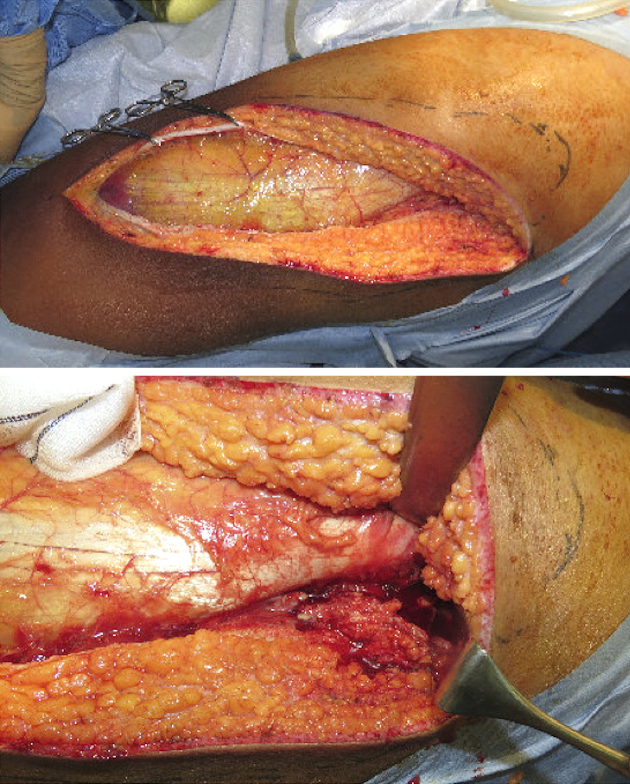
Surgical drainage and debridement of the skin and soft tissue infection of the hip and left thigh in the operating room.

A 20-year-old male student was admitted to our emergency department for dyspnea since 2 weeks. He had no previous medical and travel history. His physical examination revealed the following: Glasgow Come Scale score, E4V4M6; blood pressure, 137/83 mmHg; heart rate, 148 beats/min; respiratory rate, 40 breaths/min; and body temperature, 40.4°C. He had several abrasions on his extremities caused by playing rugby football. Initial laboratory test results of the patient are listed in Table 1. His chest radiography revealed bilateral alveolar shadows (Figure 1), and computed tomography (CT) revealed pneumonia with a cavity in the right upper lobe, which was suspected to be SPE (Figure 2A). Abdominal CT revealed SSTIs of the hip and thigh with abscesses and iliofemoral DVT (Figure 2B). Intravenous administration of vancomycin combined with clindamycin was initiated. Subsequently, he underwent surgical drainage and debridement of the SSTIs of the hip and left thigh, followed by puncture drainage of bilateral knee joint abscess in the operating room (Figure 3).

**Table 1.** Initial Results of the Patient’s Laboratory Test.

Variables	Parameter		Reference rage	
White blood cell count	31,000	cells/μL	4000–8000	cells/μL
Platelet count	11.3 × 10^4^	cells/μL	15–35 × 10^4^	cells/μL
C-reactive protein	33.1	mg/dL	<0.3	mg/dL
Fibrin/fibrinogen degradation product	17.1	μg/dL	<5.0	μg/dL
D-dimer	30	μg/mL	<0.5	μg/mL
Creatinine	1.56	mg/dL	0.60–1.20	mg/dL
Presepsin	3,445	pg/mL	<500	pg/mL

**Figure 4.**
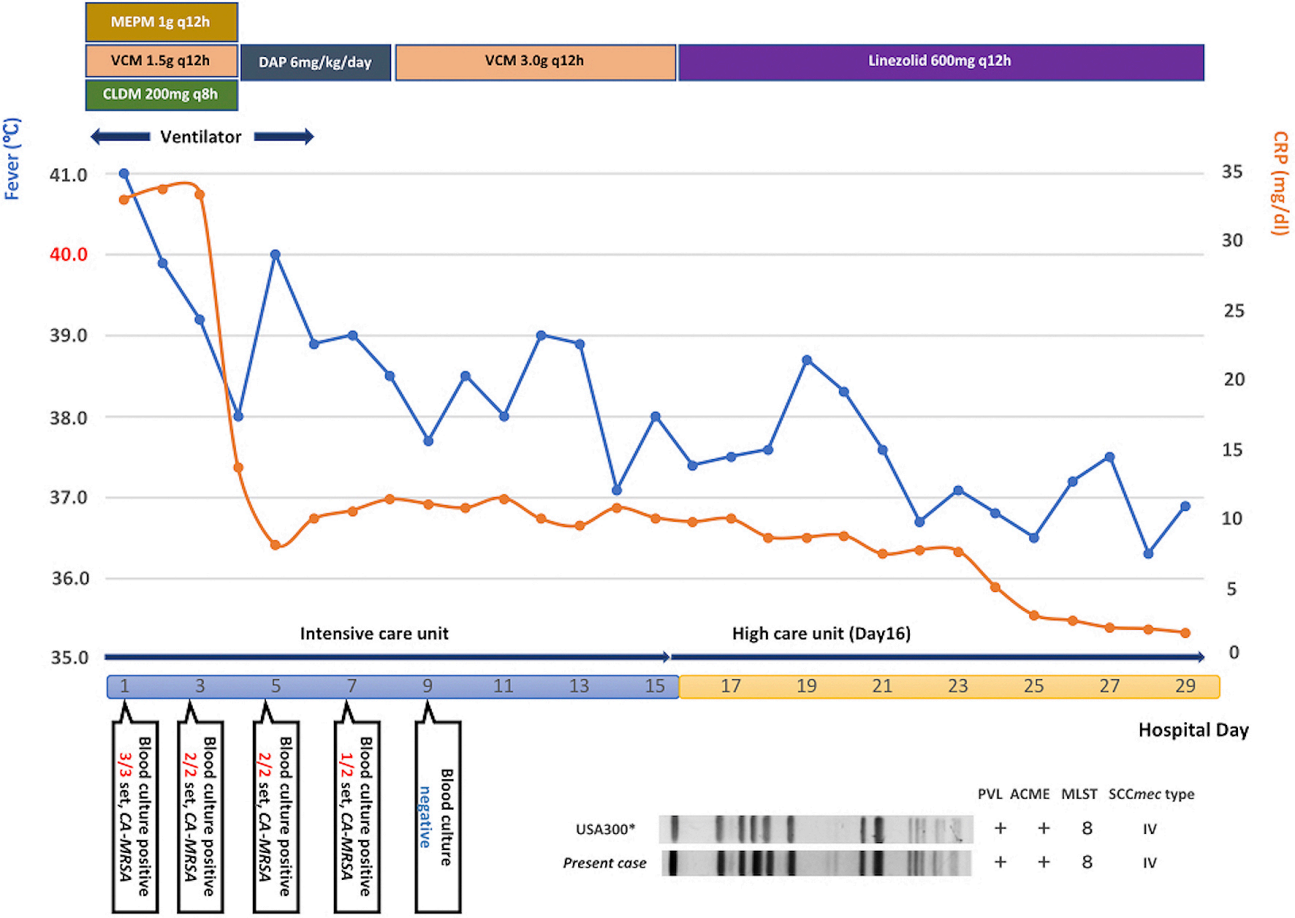
Clinical course and molecular typing of MRSA strain of the patient. *ACEM* arginine catabolic mobile element, *CA-MRSA* community-acquired methicillin-resistant *Staphylococcus aureus*, *CLDM* clindamycin, *CRP* C-reactive protein, *DAP* daptomycin, *MEPM* meropenem, *MLST * multilocus sequence typing, *PVL* Panton–Valentine leukocidin, *SCC* staphylococcal cassette chromosome, *VCM* vancomycin.

The analysis of the patient’s initial three sets of blood culture, sputum, urine, wounds, and abrasions revealed the presence of MRSA. PCR detection of virulence genes, production of PVL, SCC *mec* typing, multilocus sequence typing, and pulsed-field gel electrophoresis identified the strain as USA300 clone (Figure 4). The patient recovered from the septic shock; no further SSTIs were encountered. CT after 1 month revealed the complete disappearance of iliofemoral DVT without new pulmonary embolization, concurrent infective endocarditis, or vertebral osteomyelitis (Figure 2C). The patient was treated for septic osteomyelitis of the hip joint for 8 weeks. The patient finally became ambulatory and was discharged from our hospital after rehabilitation therapy for 11 weeks.

## Discussion

 Outbreaks of PVL-positive CA-MRSA among club teams, including American football, soccer, rugby, or wrestling teams as well as interscholastic, intercollegiate, and professional athletic teams have been reported ^(5), (6)^. Athletes are particularly vulnerable to CA-MRSA infection because of the frequency of skin injury, close-contact situations, and sharing of equipment, which is customary in athletic settings ^(7)^. The USA300 clone is widely disseminated in both community and healthcare settings, which resulted in its emergence as a worldwide pandemic clone ^(3)^. In contrast, PVL-negative ST30-IV, ST30-V, ST59-IV, ST59-V, ST89-II, and ST89-V clones are the predominant CA-MRSA strains known in Asian countries, including Japan ^(8)^. Although the prevalence of USA300 clone has been increasing in both Japanese communities and hospital settings in recent years, we could not find any report of a life-threatening infection caused by USA300 clone in Japanese athletes to date ^(4)^. The transmission route of USA300 clone in this case still remains unknown, whereas we found the report that USA300 clone was detected in 42% of nasal swabs from professional football players and staff members ^(9)^. Therefore, we strongly hypothesize that the USA300 clone of this case was derived from the nasal cavity of his teammate. To demonstrate our hypothesis, nasal screening of the collegiate rugby team is now ongoing.

SPE has a high mortality rate (<20%), and death is caused most frequently due to septic shock accompanied by multiple organ failure. It is an uncommon syndrome characterized by the embolization of an infected thrombus from a primary infectious site into the venous circulation. In the present case, sustained bacteremia lasting for 7 days was considered extremely remarkable. It was difficult to perform a surgical drainage of the deep abscess of the hip joint as a source of infection that spread to the iliofemoral vein and caused DVT. In the field of emergency medicine, we recommend for the suspicion of SSTIs as a source of SPE.

In conclusion, we raise an alarm that PVL-positive CA-MRSA, especially the USA300 clone, could be a threat among collegiate football players in Japan. Hence, we recommend the active surveillance and eradication of nasal colonization of PVL-positive CA-MRSA among collegiate athletes.

## Article Information

### Conflicts of Interest

None

### Authors’ Contributions

Conceived and designed the experiments: YR, TJ

Contributed to interpretation of data: MM, YH, KT, NH, TS, NN

Approved the final version to be submitted: MT, AT

### Approval by Institutional Review Board (IRB)

This study was approved by the ethics committee of Tokyo Medical University Hachioji Medical Center (H-232).

### Informed Consent

Written informed consent was obtained from the patient for publication of this case report and any accompanying images.
